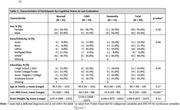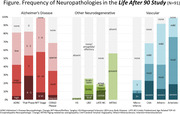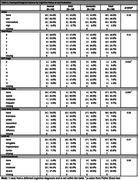# Neuropathology in the LifeAfter90 Study: 2024 update on an Ethnically Diverse Cohort Study of Oldest‐Old

**DOI:** 10.1002/alz.090552

**Published:** 2025-01-03

**Authors:** Brittany N Dugger, Lee‐Way Jin, Viharkumar Patel, Madely Martinez Pamatz, Melanie Luu, Charles Decarli, Paola Gilsanz, Dan M. Mungas, Claudia H. Kawas, María M. M. Corrada, Rachel A. Whitmer

**Affiliations:** ^1^ University of California, Davis, Sacramento, CA USA; ^2^ University of California Davis Medical Center, Davis, CA USA; ^3^ University of California, Davis, CA USA; ^4^ Kaiser Permanente Northern California Division of Research, Oakland, CA USA; ^5^ University of California, Irvine, Irvine, CA USA; ^6^ University of California, Davis School of Medicine, Sacramento, CA USA

## Abstract

**Background:**

Examining the neuropathology of the oldest‐old has significantly advanced our understanding of the multiple etiologies in very late life. Most studies have included exclusively White decedents with limited ethnoracial diversity. Our goal was to characterize neuropathology in a cohort of ethnically and racially diverse oldest‐old decedents.

**Method:**

The LifeAfter90 study is an ongoing cohort study of Kaiser Permanente Northern California members, aged 90+ with targeted recruitment of individuals across different racial/ethnic groups with no prior diagnosis of dementia in their medical record. Interviews and cognitive assessments occur approximately every 6 months. Brain donation was available to all interested consenting participants. Neuropathology was assessed using National Alzheimer’s Coordinating Center Neuropathology forms and NIA‐AA guidelines diagnoses.

**Result:**

As of January 2024, 340 participants (39%) have enrolled in autopsy (22% Asian, 18% African American, 17% Latino, 9% Multiracial/Other, and 34% White). Of the 340 participants, 91 had died and neuropathological evaluations were completed. The mean age of death was 95 years (range 90‐105), 51 (56%) were female, 12 Asian, 10 Black, 16 Latino, 44 White, and 8 Multiracial/Other. At final clinical exam, 25 participants had dementia (28%), 24 had cognitive impairment no dementia (CIND, 42%), and 41 had normal cognition (45%) (Table 1). Alzheimer disease (AD) and vascular pathologies were the most frequent findings (Figure & Table 2). Nineteen percent of participants did not have AD, one lacked neurofibrillary tangles (NFT). The most severe distributions/densities of NFTs and plaques were infrequent, with high likelihood of AD in only four participants. For vascular pathologies, 54% had moderate/severe white matter rarefaction and 76% had moderate/severe arteriolosclerosis. Thirty‐three percent had Lewy bodies, with six cases having diffuse type. Two cases had hippocampal sclerosis. One case had pathologic evidence of progressive supranuclear palsy and had dementia at last diagnosis.

**Conclusion:**

This ethnoracially diverse cohort of over 90 oldest‐old individuals reveal numerous brain pathologies are present with advanced age, with AD and select vascular pathologies being the most common. This confirms previous findings that with increases in cognitive impairment there is increasing pathology severity especially for AD pathologies.